# Challenges to Schistosomiasis Control Program in Brazil: setbacks in the control program and critical analysis of the disease notification

**DOI:** 10.1590/0037-8682-0598-2023

**Published:** 2024-07-29

**Authors:** Diogo Tavares Cardoso, Fernanda do Carmo Magalhães, Martin Johannes Enk, Stefan Michael Geiger, David Soeiro Barbosa

**Affiliations:** 1 Instituto de Ciências Biológicas, Universidade Federal de Minas Gerais, Departamento de Parasitologia, Belo Horizonte, MG, Brasil.; 2 Universidade Federal de Minas Gerais, Faculdade de Medicina Veterinária, Departamento de Medicina Veterinária Preventiva, Belo Horizonte, MG, Brasil.; 3 Ministério da Saúde, Instituto Evandro Chagas, Secretaria de Vigilância em Saúde, Ananindeua, PA, Brasil.

**Keywords:** Schistosoma mansoni, Public Health, Epidemiology, Schistosomiasis Surveillance and Control Program, Brazil

## Abstract

**Background:**

In 1970, Brazil implemented the Schistosomiasis Control Program (PCE, Portuguese acronym for *Programa de Controle da Esquistossomose*) was implemented in Brazil, where, through successive treatment interventions, the epidemiology and transmission of schistosomiasis have changed significantly over time. This study aimed to evaluate the PCE’s effectiveness by critically analyzing the disease notification system.

**Methods:**

An ecological study was conducted using data on reported schistosomiasis cases in Brazil between 2007 and 2020.

**Results:**

The highest number of municipalities actively participating in the PCE was 750, recorded in 2007. Conversely, participation reached its lowest point in 2020, with only 259 municipalities involved. Over the past decade, there has been a drastic decline in the number of municipalities with active schistosomiasis control programs. During the same period, there was an observed increase in the number of deaths caused by schistosomiasis, while the number of reported cases decreased. This suggests an inverse correlation.

**Conclusions:**

The present data suggest that schistosomiasis cases are not correctly diagnosed or reported, reflecting a twisted image of the magnitude of this public health problem in Brazil.

## INTRODUCTION

Schistosomiasis is a widespread parasitic disease affecting approximately 252 million people, reaching 54 countries, mainly Africa and Asia. These estimates suggest that roughly 770 million people live in areas where the disease is endemic and are at risk of infection^1^ ^,^ ^2^. In Brazil, intestinal schistosomiasis remains an public health problem, infecting approximately 1.5 million people with an additional 25 million living in at-risk areas^3^ ^,^ ^4^. 

The Global Burden of Disease Study (2017) estimated that schistosomiasis infections caused 1.4 million disability-adjusted life years (DALYs) in Brazil. This ranked schistosomiasis as the second-leading neglected tropical disease in the country in terms of DALYs^5^ ^,^ ^6^. Furthermore, schistosomiasis was the second most commonly reported parasitic disease in Brazil between 2009 and 2013^7^.

Brazil faces a major public challenge due to schistosomiasis, with a significant number of severe and fatal cases^1^, particularly concentrated in the Northeast and Southeast regions^8^. Endemic areas with historically high infection rates were primarily located in the states of Minas Gerais and Bahia. However, the implementation of the Brazilian Schistosomiasis Control Program (PCE Portuguese acronym for *Programa de Controle da Esquistossomose*), and ongoing treatment interventions have significantly altered the epidemiology and transmission patterns of schistosomiasis^8^ ^-^ ^10^. 

The PCE operates in a decentralized manner, with actions planned and organized at the municipal level, and mandated for inclusion in the basic healthcare plan^8^ ^,^ ^11^ ^,^ ^12^. Brazil utilizes two notification systems for schistosomiasis cases: in endemic areas are registered in the Information System of the Schistosomiasis Surveillance and Control Program (SISPCE) and severe cases are registered in the Information System of the Notification of Diseases (SINAN). In non-endemic areas all cases are registered in the SINAN^8^. This study aims to analyze the effectiveness of the national control program by critically evaluating the notification system, considering the temporal distribution of cases, severe cases, and deaths due to schistosomiasis in Brazil between 2007 and 2020.

## METHODS

### ● Ethical aspects

This Project was approved by the Brazilian Ethical Committee of Research (CEP) under license #38663720.3.0000.5149.

### ● Study area and epidemiological design

We conducted an ecological descriptive study of schistosomiasis cases reported in Brazil between 2007 and 2020. Data from the secondary database of the National Surveillance System for Notifiable Diseases of the Brazilian Ministry of Health (SINAN - *Sistema de Informação de Agravos de Notificação*) and the SISPCE were used. Data were grouped by year to examine temporal trends in schistosomiasis prevalence, deaths, and severe cases.

The PCE implements field activities to control the disease, while the SISPCE stores schistosomiasis data and supports PCE management. The 2007-2020 period was chosen because it coincides with a change in SINAN, allowing for synchronized data collection between the two systems. 

Severe cases were defined as those presenting with acute, other, or hepatosplenic forms. Intestinal and hepatointestinal forms were classified as non-severe^8^. This classification follows the Brazilian Ministry of Health’s recommendations and aligns with the notification form, where “other forms” encompass neuroschistosomiasis and any other atypical presentations. 

For SISPCE data, we calculated an annual positivity rate by dividing the total number of positive cases in each year by the total number of individuals examined. The lethality rate, using only SINAN data, was calculated by dividing the total number of schistosomiasis-related deaths by the total number of positive cases in the corresponding year.

Spearman’s rank correlation coefficients were used to assess correlations between quantitative variables. Statistical analyses were performed using SPSS 25 software.

## RESULTS

During the study period, we identified 1,095 Brazilian municipalities actively participating in the PCE. These municipalities were located in the southeastern, northeastern, southern, and northern regions of the country. It is important to note that some states, like Santa Catarina and Rondônia, only provided control program data for two years.

**FIGURE 1:**
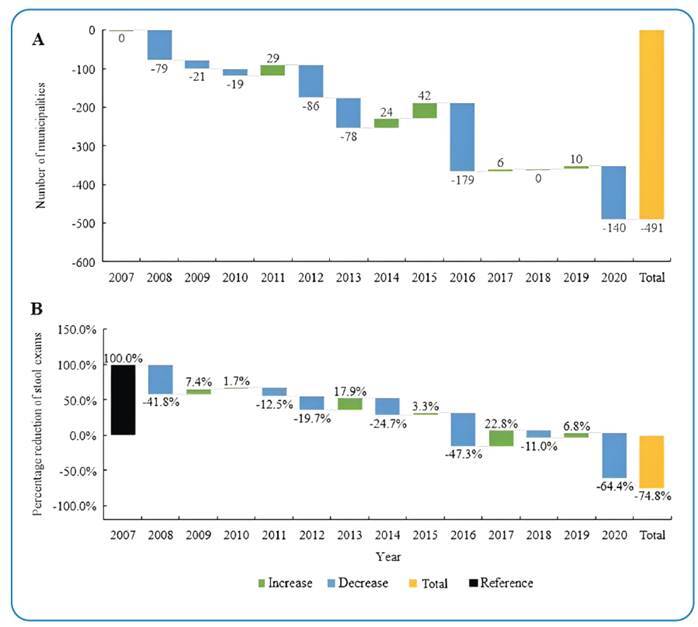
(A) Annual changes in the total number of municipalities with an active schistosomiasis control program, Blue bars indicate a decrease, and green bars indicate an increase compared to the previous year. (B) Annual percentage change in the number of stool examinations compared to the previous year. Blue bars indicate a decrease, and green bars indicate an increase.

While 1,095 municipalities participated in the program overall, their involvement decreased over time. The highest participation was 750 was observed in 2007, followed by a gradual decrease to 259 by 2020. The year 2016 saw the largest drop in participation, with 179 fewer municipalities compared to the previous year. However, there were fluctuations in this trend, with increases in participation observed in 2009, 2014, 2015, 2017, and 2019 (Table 1 and Figure 1A).

**TABLE 1:** Number of Brazilian municipalities actively participating in the Schistosomiasis Control Program (PCE) and schistosomiasis positivity rates from 2007 to 2020. Data are presented by numbers are separated by region, state, and year.

		2007		2008		2009		2010		2011		2012		2013		2014		2015		2016		2017		2018		2019		2020	
		%	MC	%	MC	%	MC	%	MC	%	MC	%	MC	%	MC	%	MC	%	MC	%	MC	%	MC	%	MC	%	MC	%	MC
North	Rondônia	0.5	8	0.0	3	0.0	0	0.0	0	0.0	0	0.0	0	0.0	0	0.0	0	0.0	0	0.0	0	0.0	0	0.0	0	0.0	0	0.0	0
	Pará	1.7	6	1.2	7	1.1	5	2.0	5	3.6	6	2.7	3	7.4	1	2.6	1	0.5	1	0.2	1	0.0	0	0.0	0	0.0	0	0.0	0
North East	Maranhão	4.8	45	4.4	36	6.4	41	5.1	40	4.6	40	5.1	35	5.0	33	3.8	33	4.1	35	2.9	32	7.5	25	6.6	28	5.8	25	5.9	18
	Piauí	0.1	3	0.0	1	0.0	0	0.0	1	0.2	1	0.0	0	0.0	0	0.0	0	0.0	0	0.0	0	0.0	0	0.0	0	0.0	0	0.0	0
	Ceará	0.3	30	0.3	25	0.2	24	0.3	25	0.1	36	0.1	37	0.2	23	0.5	27	0.4	22	0.3	9	0.1	13	0.4	11	0.1	12	0.1	9
	Rio Grande do Norte	4.3	15	2.8	14	6.9	12	2.6	16	4.6	15	3.1	14	2.1	15	4.3	15	2.0	10	2.4	6	2.0	10	1.8	12	2.0	11	1.4	5
	Paraíba	4.8	20	6.4	16	13.8	17	7.4	13	6.6	13	6.6	10	8.4	11	10.1	9	0.0	0	0.0	0	0.0	0	0.0	0	0.0	0	0.0	0
	Pernambuco	9.8	45	8.5	49	9.6	53	7.2	55	6.9	57	5.0	53	4.7	57	3.2	108	2.8	111	2.2	111	2.4	100	1.9	102	1.9	97	1.4	61
	Alagoas	7.9	63	8.0	62	9.3	63	6.3	63	6.8	65	5.8	62	5.5	64	5.3	62	4.3	60	3.9	61	4.2	60	3.5	61	3.8	59	2.4	53
	Sergipe	14.5	48	12.4	39	6.9	41	10.4	42	7.2	45	9.2	22	5.4	29	5.5	33	9.2	36	15.2	31	6.2	27	4.0	33	4.4	28	7.3	17
	Bahia	4.1	105	4.5	98	2.7	105	4.5	93	2.8	118	4.0	110	2.3	96	3.2	88	3.0	84	2.5	61	2.9	90	2.6	106	2.4	127	3.7	70
Southeast	Minas Gerais	4.8	297	4.7	265	4.1	244	3.8	228	3.8	221	3.3	189	2.3	141	2.5	116	3.1	175	2.3	47	2.5	43	1.0	22	2.0	26	1.2	17
	Espírito Santo	3.7	47	2.8	43	3.6	36	2.9	41	3.3	41	2.6	39	2.5	26	3.1	28	3.0	28	4.3	24	3.5	21	3.5	14	3.5	14	2.6	9
	Rio de Janeiro	0.4	3	0.7	1	2.8	1	0.7	2	12.6	1	0.0	0	0.0	0	0.0	0	0.0	0	0.0	0	0.0	0	0.0	0	0.0	0	0.0	0
South	Paraná	1.3	13	2.1	11	13.7	8	3.4	7	2.6	1	0.0	0	0.0	0	0.0	0	0.0	0	0.0	0	0.0	0	0.0	0	0.0	0	0.0	0
	SantaCatarina	0.3	2	0.1	1	0.0	0	0.0	0	0.0	0	0.0	0	0.0	0	0.0	0	0.0	0	0.0	0	0.0	0	0.0	0	0.0	0	0.0	0
**Total**		**5.4**	**750**	**5.2**	**671**	**5.2**	**650**	**4.7**	**631**	**4.5**	**660**	**4.2**	**574**	**3.6**	**496**	**3.7**	**520**	**3.5**	**562**	**3.2**	**383**	**3.3**	**389**	**2.9**	**389**	**2.8**	**399**	**2.5**	259

MC: Number of municipalities covered; %: Positivity rate for *S. mansoni.*

Compared to the previous year, 2020 saw the sharpest decline in the number of stool samples examined, with a decrease of 64.4% compared to 2019. The second-largest reduction occurred in 2016, with a decrease of 47.3% (Figure 1B).

**FIGURE 2:**
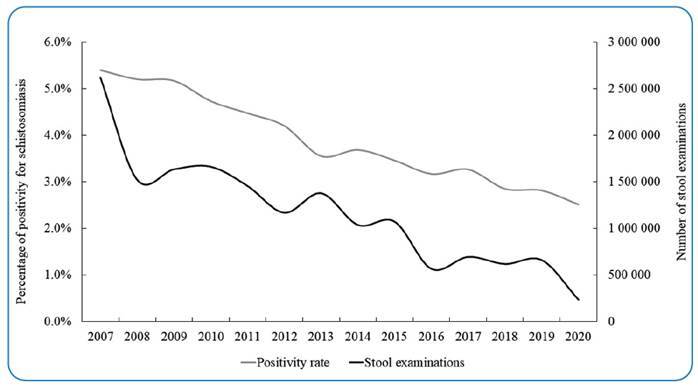
Annual positivity rate for schistosomiasis and total number of stool examinations performed annually in municipalities with an active schistosomiasis control program.

The decline mirrored a downward trend in the positivity rate for schistosomiasis among the examined population. In 2007, Brazil had a positivity rate of 5.40% (95% CI, 5.27-5.53), with roughly two million individuals examined. By 2020, the positivity rate dropped to 2.83% (95% CI, 2.75-2.93), with only 268,659 examined individuals (Supplementary Material and Figure 2).

**FIGURE 3:**
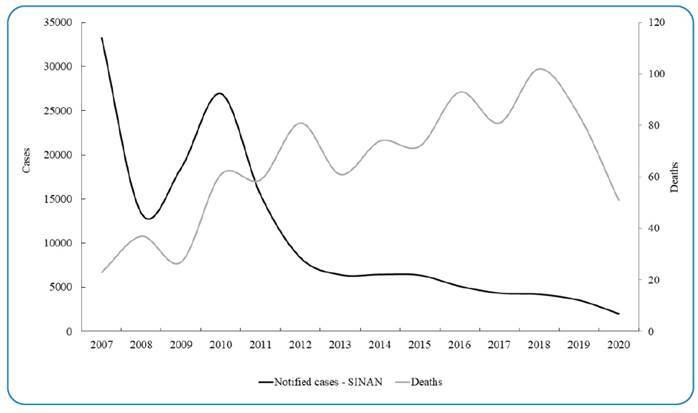
Annual number of notified schistosomiasis cases (black line) and deaths attributed to schistosomiasis (grey line) in Brazil (data from SINAN).

**FIGURE 4:**
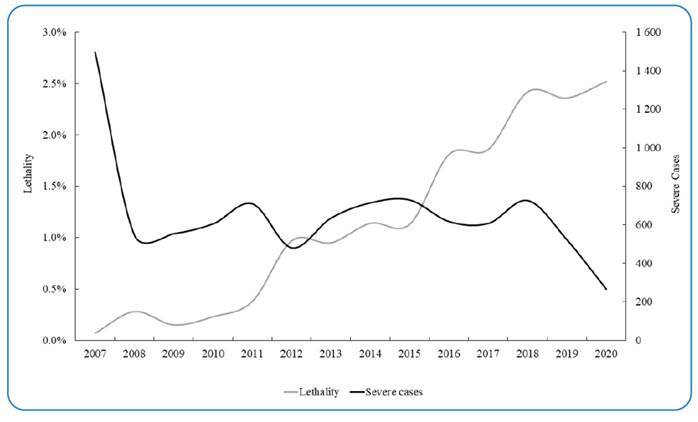
Annual number of severe schistosomiasis cases (black line) and lethality rate (grey line) in Brazil (data from SINAN only).

In addition to PCE data, we analyzed and compared data from the SINAN for the same period. During this timeframe, SINAN reported 155,008 cases. While the number of notified cases decreased over time, the graph (Figure 3) indicates an increase in the number of deaths. This resulted in a significant negative correlation (r = -0.686, *p* <0.05) between notified cases and deaths during the study period. This suggests a decrease in the total number of cases, but with a higher proportion resulting in death, leading to an increase in mortality from less than 0.1% in 2007 to approximately 2.5% in 2020. Interestingly, the number of severe cases reported by SINAN remained relatively constant throughout the period, with a total of 7,697 reported cases (Figure 4).

## DISCUSSION

This study examined the effects of the PCE in Brazil from 2007 to 2020. While our results show a decrease in schistosomiasis cases over time, a trend observed in both SISPCE and SINAN data, there’s a concerning aspect regarding PCE performance. 

Apesar do sucesso anterior relatado do programa, no Brasil houve uma diminuição nas taxas de positividade ao longo do tempo. No entanto, isto não foi acompanhado por uma diminuição no número de casos graves, resultando num aumento de mortes. The concerted actions of the PCE managed to reducing case numbers through active case finding and treatment^13^, there was a decrease in PCE activity over time, as evidenced by fewer participating municipalities and stool examinations. This raises the possibility that some municipalities may have discontinued their control programs. Potential reasons for program discontinuation could be twofold: i) the perception of a reduction in infection rates, thus prioritizing other health activities, and ii) errors in the notification of the disease.

The decrease in schistosomiasis positivity rate, from 5.4% in 2007 to 2.5% in 2020, suggests success for the PCE. However, this positive trend coincides with a decline in active case-finding efforts, as evidenced by the drop in stool examinations performed (from 2,620,752 in 2007 to 661,497 in 2019, a decrease exceeding 74%). 

This reduction might be attributed to a few factors. Inconsistencies in notification systems exist, with discrepancies between the Schistosomiasis Surveillance and Control Program Information System (SISPCE for its initials in Portuguese) and SINAN. Additionally, some municipalities might have under-reported cases as individuals treated in primary healthcare may not consistently inform the PCE team, leading to inaccuracies. This lack of communication between teams makes it difficult to report cases accurately^14^. 

The year 2020 saw a particularly sharp decline in stool tests, nearly 50% lower than 2019. This can likely be attributed to the impact of the coronavirus disease-2019 (COVID-19) pandemic. Many professionals were redeployed to combat the virus’ spread. Furthermore, the World Health Organization recommended suspending active search activities, mass treatment campaigns, and health education initiatives for neglected tropical diseases during the pandemic’s peak^15^. This recommendation likely contributed to the significant drop in stool examinations in 2020. However, concerns have been raised that interrupting control programs for *Schistosoma mansoni* and *S. haematobium* could lead to a resurgence of cases in the future^16^.

Following decades of control interventions and the implementation of the PCE, areas with low schistosomiasis endemicity have been increasingly common^10^ ^,^ ^17^. The Kato-Katz method remains the primary diagnostic tool used in control programs, even in these low-endemicity settings, due to its practicality and affordability^18^ ^,^ ^19^ However, a significant drawback of this method in these areas is its reduced sensitivity, particularly for individuals with low parasite burden^20^ ^,^ ^21^. Increasing the number of stool samples examined per slide can improve test sensitivity^20^ ^-^ ^22^. 

Recent PCE data might suggest a successful program nearing eradication. However, studies using more samples or more sensitivity tests have shown a positivity rate 2.3 times higher^21^. Despite recommendations to increase sample numbers, technical guidelines suggest increasing the number of slides examined per stool sample in low-endemicity areas^8^. However, resource limitations, both financial and human, often hinder the implementation of this approach, making it difficult to identify positive cases in areas with low endemicity or parasite burden^21^ ^,^ ^23^.

Underreporting by municipalities may be another factor contributing to the decrease in reported positive cases. Brazil utilizes two independent notification systems for schistosomiasis: SISPCE and SINAN. This dual system can lead to confusion and underreporting, potentially impacting testing, diagnosis, and notifications. Technical guidelines for schistosomiasis surveillance in Brazil recommend notifying SINAN of all diagnosed cases in. non-endemic areas, regardless of severity. Conversely, in endemic areas, only severe clinical forms should be reported to SINAN, with other cases reported to SISPCE^8^. Additionally, operational data from coproscopic, epidemiological, and malacological surveys are also included in SISPCE reports^8^. 

Rondônia is not endemic for schistosomiasis. Despite historical records suggesting its potential endemicity since the 1980s^24^, active PCE programs successfully controlled the decrease until 2008. At that point, the program was discontinued due to the presumed eradication of transmission within the state. However, reported outbreaks were not caused by local transmission but rather by infected individuals migrating from highly endemic states in southeastern or northeastern Brazil^25^. This migration risk extends beyond Rondônia, as evidenced by reports of focal transmission in Rio Grande do Sul^26^. These cases highlight the importance of continuous schistosomiasis monitoring. The absence of schistosomiasis data for São Paulo can be explained by their independent PCE notification system. This utilized its system to track program activities, rendering data incompatible with the national system^8^.

The observed negative correlation between deaths and reported cases suggests a potential consequence of reduced PCE activity. Ideally, trends in severe cases and deaths would mirror trends in reported cases; however, this was not the case in this study. The rise in lethality and continued persistence of severe cases might indicate delayed diagnosis. This, as reported elsewhere, could lead to worsening clinical situations for patients and an increased risk of death during chronic infection^16^ ^,^ ^27^. Additionally, these more severe cases could place a significant burden on the healthcare system due to increased public spending on treatment and patient follow-up.

While data shows a decline in schistosomiasis-positive cases and positivity rates over time, the number of severe cases remained relatively constant between 2008 and 2018, exceeding 500 cases annually. This suggests a potential underdiagnosis of asymptomatic cases or a lack of proper case management. The decrease in severe cases observed in 2019 and 2020 could be attributed to several factors, including the COVID-19 pandemic, competing healthcare priorities, or delays in reporting^16^. 

Furthermore, the suspected decline in PCE activity in many municipalities is corroborated by the observed increase in deaths and lethality during the study period. This trend coincides with a strong negative correlation between reported positive cases and deaths in the SINAN data.

While schistosomiasis is chronic with a slow progression, the leading causes of death are often related to severe clinical forms like liver cirrhosis, portal hypertension, colitis, pulmonary complications, and neurological issues. These conditions typically develop decades after the initial infection^25^ ^,^ ^28^. The observed rise in lethality, coupled with the negative correlation between lethality and reported cases, suggests potential underreporting or misdiagnosis. Both scenarios are concerning and could represent a setback in Brazil’s fight against schistosomiasis.

The latest National Survey of the Prevalence of Schistosomiasis Mansoni and Soil-Transmitted Helminth Infections (2010-2015) estimated a national schistosomiasis prevalence of 0.9% in Brazil (Katz, 2018). However, this school-based survey had limited coverage in key endemic areas, particularly southeastern and northeastern regions. Additionally, the results weren’t statistically adjusted to account for the reduced sensitivity of the two-slide Kato-Katz method, as documented in other studies^20^ ^,^ ^21^ ^,^ ^29^. This potentially misleading data might have influenced health policymakers, leading to the downplay of the schistosomiasis problem in Brazil. Consequently, control efforts and financial support for municipal interventions may have been reduced. 

A false perception of nearing schistosomiasis elimination, combined with the discontinuation of control activities, could result in a resurgence of the disease in Brazil, as there are still areas with active transmission in several states of the country^30^ ^,^ ^31^. If control activities are not resumed, there may be a significant increase in schistosomiasis infection rates^12^. This has already been reported, and the situation might worsen due to the interruption of neglected tropical disease control programs during the COVID-19 pandemic^27^ ^,^ ^32^ ^,^ ^33^.

Schistosomiasis is a chronic, debilitating disease that imposes a significant burden on the Brazilian public health system. The recent decline in PCE activity coincides with an increase in reported schistosomiasis lethality, leading to higher in public healthcare costs^34^. In most endemic areas, control primarily relies on diagnosis and preventive chemotherapy for at-risk populations. Therefore, continued PCE efforts are crucial for disease control and achieving the Sustainable Development Goals outlined in the 2030 agenda^35^ ^,^ ^36^.

As this study relied on secondary data, limitations exist regarding data availability and utilization. Endemic areas might have issues with incomplete reporting, leading to missing or unknown schistosomiasis information. Additionally, the ecological study design prevents us from establishing causal relationships. Despite these limitations, the data provided statistically significant insights. It revealed a concerning decline in PCE activity, jeopardizing Brazil’s goal of eliminating schistosomiasis as a public health issue by 2030^35^.

This decline in PCE activity poses serious public health threats. Without the program’s early diagnosis efforts, the number of severe cases increases, leading to higher healthcare system costs. The gradual decrease in control activities over the past five years has reached worrying levels. It hinders early diagnosis, potentially leading to an expansion of endemic areas, a rise in severe cases, and a significant financial burden on the health sector.

## References

[1] Colley DG, Bustinduy AL, Secor WE, King CH (2014). Human schistosomiasis. The Lancet.

[2] World Health Organization (WHO) (2023). schistosomiasis (Bilharzia).

[3] Graeff-Teixeira C, Pieri OS (2022). Schistosomiasis Control: Present Situation and Perspectives.

[4] Noya O, Katz N, Pointier JP, Franco-Paredes C, Santos-Preciado JI (2015). Neglected Tropical Diseases.

[5] GBD 2017 DALYs and HALE Collaborators (2018). Global, regional, and national disability-adjusted life-years (DALYs) for 359 diseases and injuries and healthy life expectancy (HALE) for 195 countries and territories, 1990-2017: a systematic analysis for the Global Burden of Disease Study 2017. Lancet.

[6] Global Burden of Disease Study (GBD) (2021). Schistosomiasis - Level 3 cause | Institute for Health Metrics and Evaluation.

[7] Brandão E, Romero S, da Silva MAL, Santos FLN (2017). Neglected tropical diseases in Brazilian children and adolescents: Data analysis from 2009 to 2013. Infect Dis Poverty.

[8] Brasil (2014). Vigilância da Esquistossomose Mansoni - Diretrizes técnicas.

[9] Scholte RGC, Gosoniu L, Malone JB, Chammartin F, Utzinger J, Vounatsou P (2014). Predictive risk mapping of schistosomiasis in brazil using bayesian geostatistical models. Acta Trop.

[10] Katz N, Katz N (2018). Inquérito Nacional de Prevalência da Esquistossomose mansoni e Geo-helmintoses.

[11] Souza MDR, De Jesus DMS, Santos AHC, Lima SVMA, Dos Santos A, Tavares DDS (2022). Risk clusters of Schistosoma mansoni infection in an endemic state of Brazil: space-time modelling and association with socio-economic and environmental factors. Trans R Soc Trop Med Hyg.

[12] Cruz JIN, Salazar G de O, La Corte R (2020). Retrocesso do Programa de Controle da Esquistossomose no estado de maior prevalência da doença no Brasil. Rev Panamazonica Saude.

[13] Amaral RS do, Tauil PL, Lima DD, Engels D (2006). An analysis of the impact of the Schistosomiasis Control Programme in Brazil. Mem Inst Oswaldo Cruz.

[14] Quites HFDO, Abreu MNS, Matosoi LF, Gazzinelli A (2016). Evaluation of schistosomiasis control activities in the Family Health Strategy in municipalities in the Jequitinhonha Valley, Minas Gerais, Brazil. Revista Brasileira de Epidemiologia.

[15] WHO (2020). Community-based health care, including outreach and campaigns, in the context of the COVID-19 pandemic Interim guidance.

[16] Kura K, Ayabina D, Toor J, Hollingsworth TD, Anderson RM (2021). Disruptions to schistosomiasis programmes due to COVID-19: An analysis of potential impact and mitigation strategies. Trans R Soc Trop Med Hyg.

[17] Drummond SC, Pereira SRS, Silva LC dos S, Antunes CMF, Lambertucci JR (2010). Schistosomiasis control program in the state of Minas Gerais in Brazil. Mem Inst Oswaldo Cruz.

[18] Katz N, Chaves A, Pellegrino J (1972). A simple device for quantitative stool thick-smear technique in Schistosomiasis mansoni. Rev Inst Med Trop Sao Paulo.

[19] World Health Organization (WHO) The control of schistosomiasis: Report of a WHO Expert Committee.

[20] Enk MJ, Lima ACL, Drummond SC, Schall VT, Coelho PMZ (2008). The effect of the number of stool samples on the observed prevalence and the infection intensity with Schistosoma mansoni among a population in an area of low transmission. Acta Trop.

[21] Oliveira WJ, Magalhães F do C, Elias AMS, de Castro VN, Favero V, Lindholz CG (2018). Evaluation of diagnostic methods for the detection of intestinal schistosomiasis in endemic areas with low parasite loads: Saline gradient, Helmintex, Kato-Katz and rapid urine test. PLoS Negl Trop Dis.

[22] Pinheiro MCC, Carneiro TR, Hanemann AL de P, Oliveira SM de, Bezerra FSM (2012). The combination of three faecal parasitological methods to improve the diagnosis of schistosomiasis mansoni in a low endemic setting in the state of Ceará, Brazil. Mem Inst Oswaldo Cruz.

[23] Lindholz CG, Favero V, Verissimo C de M, Candido RRF, de Souza RP, dos Santos RR, Lamberton PHL (2018). Study of diagnostic accuracy of Helmintex, Kato-Katz, and POC-CCA methods for diagnosing intestinal schistosomiasis in Candeal, a low intensity transmission area in northeastern Brazil. PLoS Negl Trop Dis.

[24] Coimbra CE, Santos RV, Smanio L (1984). Potencial endêmico da esquistossomose para o Estado de Rondônia, Brasil. Rev Saude Publica.

[25] Pereira AD, Pinto PLS, Camargo J de SAA, de Souza JBR, Amante CA, de Souza VKG (2016). Potential for shistosomiasis in a municipality of Rondônia, Brazilian Amazon. Acta Amazon.

[26] Ramírez A da P, Favero V, Lindholz CG, Veríssimo C de M, Pascoal VF, Candido RRF (2020). Schistosomiasis: an epidemiological update on Brazil’s southernmost low endemic area in Esteio. Rev Soc Bras Med Trop.

[27] Dantas NM, Andrade LA, Paz WS da, Borges WN, Barbosa VGB, Hora DPG da (2023). Impact of the COVID-19 pandemic on the actions of the Schistosomiasis Control Program in an endemic area in Northeastern Brazil. Acta Trop.

[28] Maroja RC (1953). Incidência de esquistossomose em Fordlândia, município de Itaituba, estado do Pará. Revista do Serviço Especial de Saúde Pública.

[29] Pinheiro MCC, Carneiro TR, Hanemann AL de P, Oliveira SM de, Bezerra FSM (2012). The combination of three faecal parasitological methods to improve the diagnosis of schistosomiasis mansoni in a low endemic setting in the state of Ceará, Brazil. Mem Inst Oswaldo Cruz.

[30] Bezerra D de F, Pinheiro MCC, Barbosa L, Viana AG, Fujiwara RT, Bezerra FS de M (2021). Diagnostic comparison of stool exam and point-of-care circulating cathodic antigen (POC-CCA) test for schistosomiasis mansoni diagnosis in a high endemicity area in northeastern Brazil. Parasitology.

[31] Graeff-Teixeira C, Favero V, Pascoal VF, de Souza RP, Rigo F de V, Agnese LHD (2021). Low specificity of point-of-care circulating cathodic antigen (POCCCA) diagnostic test in a non-endemic area for schistosomiasis mansoni in Brazil. Acta Trop.

[32] Souza M do R, Paz WS da, Sales VB dos S, Jesus GFH de, Tavares D dos S, Lima SVMA (2022). Impact of the COVID-19 Pandemic on the Diagnosis of Tuberculosis in Brazil: Is the WHO End TB Strategy at Risk?. Front Pharmacol.

[33] da Paz WS, Souza M do R, Tavares D dos S, de Jesus AR, dos Santos AD, do Carmo RF (2022). Impact of the COVID-19 pandemic on the diagnosis of leprosy in Brazil: An ecological and population-based study. The Lancet Regional Health - Americas.

[34] Nascimento GL, De Oliveira MRF (2014). Severe forms of schistosomiasis mansoni: epidemiologic and economic impact in Brazil, 2010. Trans R Soc Trop Med Hyg.

[35] World Health Organization (WHO) (2021). Ending the neglect to attain the sustainable development goals: a road map for neglected tropical diseases 2021-2030.

[36] World Health Organization (WHO) (2022). WHO guideline on control and elimination of human schistosomiasis.

