# Supplementary material for: Challenges to Schistosomiasis Control Program in Brazil: setbacks in the control program and critical analysis of the disease notification
Source: Rev Soc Bras Med Trop. 2024 Jul 29;57:e00409-2024. doi: 10.1590/0037-8682-0598-2023 (PMC11290847; doi:10.1590/0037-8682-0598-2023)
Supplement: Supplementary file 1 [file 1678-9849-rsbmt-57-e00409-2024-supp1.pdf]

Number of Brazilian municipalities by states participating in the Schistosomiasis Control Program (PCE) between 2007-2020. Data showing number of tests performed, positivity rate and number of positivity for *S. Mansoni*.

| | Estado | 2007 | | | 2008 | | | 2009 | | | 2010 | | | 2011 | | |
|---|---|---|---|---|---|---|---|---|---|---|---|---|---|---|---|---|
| | | po | % | EX | po | % | EX | po | % | EX | po | % | EX | po | % | EX |
| | Rondônia | 21 | 0,5% | 4494 | 0 | 0,0% | 447 | 0 | 0,0% | 0 | 0 | 0,0% | 0 | 0 | 0,0% | 0 |
| Noth | Pará | 405 | 1,7% | 20247 | 467 | 1,2% | 22578 | 332 | 1,1% | 22464 | 164 | 2,0% | 12919 | 448 | 3,6% | 17779 |
| | Maranhão | 5363 | 4,8% | 134078 | 3904 | 4,4% | 90640 | 5200 | 6,4% | 111879 | 5083 | 5,1% | 102260 | 3381 | 4,6% | 93640 |
| | Piauí | 4 | 0,1% | 6841 | 2 | 0,0% | 5426 | 0 | 0,0% | 0 | 1 | 0,0% | 2645 | 12 | 0,2% | 4986 |
| | Ceará | 211 | 0,3% | 62016 | 183 | 0,3% | 47718 | 140 | 0,2% | 55408 | 125 | 0,3% | 45239 | 41 | 0,1% | 31507 |
| | Rio Grande do Norte | 2326 | 4,3% | 61663 | 1405 | 2,8% | 43807 | 1807 | 6,9% | 39866 | 2829 | 2,6% | 104628 | 1073 | 4,6% | 37267 |
| | Paraíba | 6103 | 4,8% | 135487 | 4123 | 6,4% | 63924 | 6945 | 13,8% | 116438 | 2295 | 7,4% | 48960 | 2590 | 6,6% | 48835 |
| | Pernambuco | 12789 | 9,8% | 124070 | 8850 | 8,5% | 88987 | 8718 | 9,6% | 96756 | 8187 | 7,2% | 98665 | 7623 | 6,9% | 103961 |
| | Alagoas | 17597 | 7,9% | 216903 | 16203 | 8,0% | 193808 | 16321 | 9,3% | 185538 | 13283 | 6,3% | 180994 | 13003 | 6,8% | 177655 |
| | Sergipe | 13677 | 14,5% | 131235 | 8258 | 12,4% | 79858 | 8199 | 6,9% | 92957 | 8844 | 10,4% | 91719 | 8534 | 7,2% | 100387 |
| North East | Bahia | 18890 | 4,1% | 618892 | 15298 | 4,5% | 373048 | 10098 | 2,7% | 408606 | 10787 | 4,5% | 279855 | 10151 | 2,8% | 313105 |
| | Minas Gerais | 32782 | 4,8% | 981878 | 19446 | 4,7% | 411559 | 17115 | 4,1% | 427766 | 21609 | 3,8% | 598797 | 16104 | 3,8% | 451593 |
| | Espírito Santo | 3475 | 3,7% | 101306 | 2880 | 2,8% | 90070 | 2907 | 3,6% | 74880 | 2163 | 2,9% | 94931 | 1891 | 3,3% | 76316 |
| Southeast | Rio de Janeiro | 6 | 0,4% | 1962 | 2 | 0,7% | 187 | 13 | 2,8% | 348 | 5 | 0,7% | 2164 | 12 | 12,6% | 657 |
| | Paraná | 255 | 1,3% | 18933 | 225 | 2,1% | 10503 | 266 | 13,7% | 4373 | 33 | 3,4% | 1507 | 4 | 2,6% | 112 |
| South | Santa Catarina | 4 | 0,3% | 747 | 2 | 0,1% | 1539 | 0 | 0,0% | 0 | 0 | 0,0% | 0 | 0 | 0,0% | 0 |
| | Total | 113908 | 5,4% | 2620752 | 81248 | 5,2% | 1524099 | 78061 | 5,2% | 1637279 | 75408 | 4,7% | 1665283 | 64867 | 4,5% | 1457800 |

| | Estado | 2012 | | | 2013 | | | 2014 | | | 2015 | | | 2016 | | |
|---|---|---|---|---|---|---|---|---|---|---|---|---|---|---|---|---|
| | | po | % | EX | po | % | EX | po | % | EX | po | % | EX | po | % | EX |
| | Rondônia | 0 | 0,0% | 0 | 0 | 0,0% | 0 | 0 | 0,0% | 0 | 0 | 0,0% | 0 | 0 | 0,0% | 0 |
| Noth | Pará | 167 | 2,7% | 6316 | 28 | 7,4% | 241 | 25 | 2,6% | 814 | 15 | 0,5% | 2189 | 8 | 0,2% | 2437 |
| | Maranhão | 2543 | 5,1% | 93236 | 2865 | 5,0% | 81033 | 3145 | 3,8% | 82691 | 2227 | 4,1% | 69340 | 1162 | 2,9% | 45747 |
| | Piauí | 0 | 0,0% | 0 | 0 | 0,0% | 0 | 0 | 0,0% | 0 | 0 | 0,0% | 0 | 0 | 0,0% | 0 |
| | Ceará | 44 | 0,1% | 34680 | 53 | 0,2% | 27146 | 117 | 0,5% | 29581 | 126 | 0,4% | 22018 | 47 | 0,3% | 13175 |
| | Rio Grande do Norte | 837 | 3,1% | 22295 | 612 | 2,1% | 34684 | 727 | 4,3% | 25671 | 247 | 2,0% | 10960 | 213 | 2,4% | 6475 |
| | Paraíba | 2151 | 6,6% | 38010 | 2020 | 8,4% | 25694 | 2410 | 10,1% | 23616 | 0 | 0,0% | 0 | 0 | 0,0% | 0 |
| | Pernambuco | 5338 | 5,0% | 94544 | 6510 | 4,7% | 120182 | 8754 | 3,2% | 257417 | 7341 | 2,8% | 244844 | 4380 | 2,2% | 190453 |
| | Alagoas | 11384 | 5,8% | 166798 | 12652 | 5,5% | 174090 | 9775 | 5,3% | 162633 | 7965 | 4,3% | 158806 | 6507 | 3,9% | 131097 |
| | Sergipe | 1767 | 9,2% | 18070 | 3382 | 5,4% | 50614 | 3227 | 5,5% | 47058 | 4580 | 9,2% | 61485 | 2827 | 15,2% | 29512 |
| North East | Bahia | 15698 | 4,0% | 380464 | 6004 | 2,3% | 522145 | 5693 | 3,2% | 170154 | 3976 | 3,0% | 128856 | 1615 | 2,5% | 71823 |
| | Minas Gerais | 8323 | 3,3% | 251172 | 8019 | 2,3% | 304094 | 5373 | 2,5% | 198874 | 8550 | 3,1% | 343090 | 1253 | 2,3% | 57638 |
| | Espírito Santo | 1585 | 2,6% | 64842 | 860 | 2,5% | 39961 | 1114 | 3,1% | 40832 | 776 | 3,0% | 32458 | 567 | 4,3% | 18144 |
| Southeast | Rio de Janeiro | 0 | 0,0% | 0 | 0 | 0,0% | 0 | 0 | 0,0% | 0 | 0 | 0,0% | 0 | 0 | 0,0% | 0 |
| | Paraná | 0 | 0,0% | 0 | 0 | 0,0% | 0 | 0 | 0,0% | 0 | 0 | 0,0% | 0 | 0 | 0,0% | 0 |
| South | Santa Catarina | 0 | 0,0% | 0 | 0 | 0,0% | 0 | 0 | 0,0% | 0 | 0 | 0,0% | 0 | 0 | 0,0% | 0 |
| | Total | 49837 | 4,2% | 1170427 | 43005 | 3,6% | 1379884 | 40360 | 3,7% | 1039341 | 35803 | 3,5% | 1074046 | 18579 | 3,2% | 566501 |

| | Estado | 2017 | | | 2018 | | | 2019 | | | 2020 | | |
|---|---|---|---|---|---|---|---|---|---|---|---|---|---|
| | | po | % | EX | po | % | EX | po | % | EX | po | % | EX |
| | Rondônia | 0 | 0,0% | 0 | 0 | 0,0% | 0 | 0 | 0,0% | 0 | 0 | 0,0% | 0 |
| Noth | Pará | 0 | 0,0% | 0 | 0 | 0,0% | 0 | 0 | 0,0% | 0 | 0 | 0,0% | 0 |
| | Maranhão | 1871 | 7,5% | 44589 | 2608 | 6,6% | 55795 | 2818 | 5,8% | 50842 | 1094 | 5,9% | 20427 |
| | Piauí | 0 | 0,0% | 0 | 0 | 0,0% | 0 | 0 | 0,0% | 0 | 0 | 0,0% | 0 |
| | Ceará | 39 | 0,1% | 20855 | 55 | 0,4% | 18882 | 24 | 0,1% | 12792 | 22 | 0,1% | 8365 |
| | Rio Grande do Norte | 314 | 2,0% | 12184 | 201 | 1,8% | 16507 | 306 | 2,0% | 13717 | 63 | 1,4% | 3738 |
| | Paraíba | 0 | 0,0% | 0 | 0 | 0,0% | 0 | 0 | 0,0% | 0 | 0 | 0,0% | 0 |
| | Pernambuco | 4446 | 2,4% | 176176 | 3770 | 1,9% | 159810 | 3962 | 1,9% | 188877 | 838 | 1,4% | 50356 |
| | Alagoas | 7030 | 4,2% | 148242 | 5980 | 3,5% | 156609 | 5759 | 3,8% | 162770 | 2353 | 2,4% | 83298 |
| | Sergipe | 2831 | 6,2% | 40065 | 2311 | 4,0% | 43473 | 1227 | 4,4% | 20906 | 423 | 7,3% | 12099 |
| North East | Bahia | 4956 | 2,9% | 183389 | 3667 | 2,6% | 128701 | 5119 | 2,4% | 180499 | 1525 | 3,7% | 36312 |
| | Minas Gerais | 1449 | 2,5% | 55329 | 342 | 1,0% | 28265 | 283 | 2,0% | 14216 | 177 | 1,2% | 12202 |
| | Espírito Santo | 437 | 3,5% | 15034 | 438 | 3,5% | 11374 | 460 | 3,5% | 16878 | 258 | 2,6% | 8601 |
| Southeast | Rio de Janeiro | 0 | 0,0% | 0 | 0 | 0,0% | 0 | 0 | 0,0% | 0 | 0 | 0,0% | 0 |
| | Paraná | 0 | 0,0% | 0 | 0 | 0,0% | 0 | 0 | 0,0% | 0 | 0 | 0,0% | 0 |
| South | Santa Catarina | 0 | 0,0% | 0 | 0 | 0,0% | 0 | 0 | 0,0% | 0 | 0 | 0,0% | 0 |
| | Total | 23373 | 3,3% | 695863 | 19372 | 2,9% | 619416 | 19958 | 2,8% | 661497 | 6753 | 2,5% | 235398 |

EX Number of tests perfomed
% Positivity rate for *S. mansoni*
Po Number of Positivity for *S. Mansoni*
